# Illusory Essences: A Bias Holding Back Theorizing in Psychological Science

**DOI:** 10.1177/1745691621991838

**Published:** 2021-07-20

**Authors:** C. Brick, B. Hood, V. Ekroll, L. de-Wit

**Affiliations:** 1 Department of Psychology, University of Amsterdam; 2 Department of Psychology, University of Cambridge; 3 School of Psychological Science, University of Bristol; 4 Department of Psychosocial Science, University of Bergen

**Keywords:** essentialism, natural kinds, categories, labels, validity, metascience

## Abstract

The reliance in psychology on verbal definitions means that psychological research is unusually moored to how humans think and communicate about categories. Psychological concepts (e.g., intelligence, attention) are easily assumed to represent objective, definable categories with an underlying essence. Like the “vital forces” previously thought to animate life, these assumed essences can create an illusion of understanding. By synthesizing a wide range of research lines from cognitive, clinical, and biological psychology and neuroscience, we describe a pervasive tendency across psychological science to assume that essences explain phenomena. Labeling a complex phenomenon can appear as theoretical progress before there is sufficient evidence that the described category has a definable essence or known boundary conditions. Category labels can further undermine progress by masking contingent and contextual relationships and obscuring the need to specify mechanisms. Finally, we highlight examples of promising methods that circumvent the lure of essences and suggest four concrete strategies for identifying and avoiding essentialist intuitions in theory development.

Much of the confusion in experimental psychology comes from terminology. . . . “Intelligence” is simply a word from everyday language. . . . We have every reason to suspect that it will have no “real essence” at all.

—Duncan (2010, p. 26)

The history of science is replete with searches for illusory concepts and processes. For example, in biology, “vital forces” were long assumed to constitute an essence that animated life. In this article, we synthesize evidence from across psychological science to argue that essences are particularly problematic in psychology. Methodological advances have highlighted how cognitive biases can hamper scientific methods and lead to spurious conclusions (Bishop, 2020; Simmons et al., 2011). We present evidence that the intuitive appeal of essences is creating theoretical dead ends across diverse areas of psychology and provide concrete advice for theory development.

Researchers in several areas are growing concerned that the way psychologists conceptualize phenomena is leading to theoretical impasses (Barrett, 2006; Fiske, 2020). The field of developmental disorders illustrates how problems can arise from searches for core deficits that are assumed to offer a common mechanistic origin for individuals with the same diagnostic label (Astle & Fletcher-Watson, 2020). In this and other fields, psychological concepts are unlikely to be underpinned by a unitary cause (such as vital forces) but may instead emerge from interactions between factors. We argue that psychological theorizing can be improved by recognizing a cognitive bias toward essences.

## Essentialism

Categorization is the process of how ideas and objects are recognized and differentiated. *Essentialism* is the view that concepts such as tree, attention, or anger each have an underlying essence that makes them what they are. This view originates from classical Greek thinkers, including Plato and Aristotle, and the later term “quintessence,” meaning the essence that characterizes a thing. We define *psychological essentialism* as comprising two interrelated beliefs: that certain categories are discovered rather than created and that an internal essence causes the category membership and explains related mechanisms. Psychological essentialism occurs when one assumes that

certain categories are real rather than human constructions (i.e., these categories are thought to be natural, discovered, information-rich, carving nature at its joints), and that these natural categories possess an underlying causal force (the “essence”) that is responsible for category members being the way they are and sharing so many properties. (Gelman & Rhodes, 2012, p. 4; see also Hull, 1965)

This definition is closely related to placeholder essentialism (Medin & Ortony, 1989), quintessence (Leslie, 2013), natural kinds (Barrett, 2006; Wilson et al., 2007), reification, and vernacular lexemes (Fiske, 2020). As with many well-established psychological concepts (e.g., confirmation bias), the structure and mechanisms of essentializing are still being investigated. We suspect that the tendency to essentialize is an emergent property based on multiple mechanisms and processes. Indeed, empirical work has suggested that assumptions about categories can be separated into subtypes such as innate origins, internal commonalities, immutability, and stability over time (Gelman et al., 2007), and these different types may have distinct etiologies.

To gain a feeling for the effortless tendency to think in underlying essences even when there are none, consider the Ship of Theseus thought experiment. A wooden ship is replaced plank by plank. At what moment does it stop being the same ship? There is no physical paradox because there was never a unitary physical essence of the ship that pervaded its original but not replacement planks. However, the stopping decision still feels like a quandary because of the conflict between the strong intuition that the ship contains a material essence and our inability to locate this essence when the planks are replaced. This intuition is even stronger for living categories such as animals (Gelman, 2005b; Keil, 1989). For example, adults and children are more likely to consider a radically transformed object the same even when it is described as an unusual animal (starfish) as opposed to an inanimate paperweight (Hall, 1998).

Essentialist assumptions rarely hold up to scrutiny in any area of science. Even seemingly fundamental and natural categories such as hydrogen (an element with one proton) are not unitary; three different hydrogen isotopes have distinct etiologies, structures, and properties (Leslie, 2013). For psychological concepts such as memory, intelligence, attention, or depression, the tendency toward essentialism can lead to neglecting that these categories are constructs that may or may not carve nature at its joints and yet are frequently assumed to have a single cause or underlying mechanism. Labeling a category and assuming it has an underlying essence may provide an illusion of explanatory depth (Rozenblit & Keil, 2002).

## Function of Essentialism

Human cognition appears prone to essentialist intuitions from early childhood through adulthood (Gelman, 2005a; Leslie, 2013) perhaps because essentialism serves as an effective heuristic to learn and operate in the world. These intuitive theories are the basis for conceptual development across a range of domains of knowledge (Carey, 2011). For example, preschool children assume that category members share an underlying structure, that there is an innate or biological basis to their category membership, and that the categories have fixed boundaries (Gelman, 2005b; Keil, 1989). Children infer that one animal raised by a different species will prefer the food of its biological rather than its adoptive parents and that an animal does not become a different species when its external features change (e.g., painting stripes on a horse does not make it a zebra; Keil, 1989). Young children even make inferences about which clothing or jobs are female versus male (Gelman et al., 2004). There may be no universal essence to femininity, but applying the label can feel intuitive and easy. Part of the bias toward essentialist inferences may come from a tendency to regard features as inherent (Cimpian & Salomon, 2014). For example, some individuals might be biased to assume that pink is inherently feminine rather than an arbitrary cultural fashion. For scientists, these assumptions can bias reasoning and privilege intuitive but misguided explanations (Leslie, 2013).

This tendency to essentialize is one example of how minds do not just passively absorb sensory data but apply assumptions that organize and interpret sensations. This point has long been recognized in Western philosophy, such as in Kant’s critique that reasoning does not exist independently of how minds are organized to construct meaning (Kant, 1855). These cognitive processes can lead to systematic problems when building and testing scientific theories (Bishop, 2020; Henrich et al., 2010; Oberauer & Lewandowsky, 2019). In particular, previous evidence has shown that essentialism interferes with scientific thinking about biological categories such as species (Gelman et al., 2004), broad theories such as evolution and natural selection (Gelman & Rhodes, 2012; Shtulman & Schulz, 2008), and psychological constructs such as emotions (Barrett, 2017). The key aims of the current article are to demonstrate that essentialist thinking may pervade theory building across more psychological fields than was previously recognized. We suggest that psychologists would benefit from explicit strategies to mitigate these tendencies.

Scientific advances in other fields have emerged curiously often from rejecting an earlier account built on illusory essences. Before Darwin, the dominant contemporary assumption was that each species belonged to a separate, fixed category with a unique essence. Historians of science have also identified essentialist beliefs as a major impediment to the discovery of natural selection (Shtulman & Schulz, 2008). Essentialist thinking about species is still common today (Barrett, 2017). Darwin overcame this assumption by documenting variability (e.g., in beak sizes) and context (e.g., food sources on different islands; Mayr, 1963). Moving past the essence assumption ultimately allowed Darwin to unearth the mechanisms of natural selection that drive variability across species. Below, we explore the two key errors of essentialism (Gelman & Rhodes, 2012) and their consequences for scientific reasoning.

## Assumed Natural Kinds

The first assumption in essentialism is that observed categories are discovered rather than constructed. This assumption may be paired with a related belief that any given category carves nature at its joints—that the category is natural and an appropriate and informative separation of the components of a phenomenon. Some information-processing systems (e.g., artificial intelligence) might struggle to operate on terms such as female, attention, or face without conceptual constraints for what defines those categories and their boundaries. In contrast, humans are quick to label assumed categories, many of which do not have definable essences. This aspect of language use was famously illustrated by a challenge to define what all games have in common (Wittgenstein, 1953). The meaning of game feels intuitive and effortless, but it is surprisingly difficult to define in a way that specifies the shared attributes of diverse activities such as chess, role playing, and football, except by using additional categories that also rely on undefined essences. “Game” ultimately refers to a network of semantic and contextual associations and serves a functional rather than precise definitional role; it does not have a defined essence with consistent boundaries. With psychological constructs, essentialism can lead to prematurely closing off research to variability across contexts or over time. For example, rapid-eye-movement (REM) sleep has very different characteristics across species and development, and therefore the label REM can be misleading. As pointed out by Blumberg, “It is when we look to the diversity of sleep across species, ages, and environmental contexts that the inadequacies of current research conventions most clearly present themselves” (Blumberg et al., 2020, p. R38).

## Assumed Internal Causes

The second key assumption is that these putative natural kinds possess an underlying causal force—the essence—that drives category membership and its properties. This placeholder essentialism (Medin & Ortony, 1989) can occur whether or not one can define the essence or the boundary conditions. This assumption appears to affect cognition across a range of domains from how children reason about biology (Keil, 1989) to how adults form stereotypes (Yzerbyt et al., 1997). A clear example in adults is the tendency to assume that heritable individual differences are deterministically caused by single genes (Dar-Nimrod & Heine, 2011).

Combining both assumptions—natural kinds and internal causes—could help to explain why certain kinds of pseudoscience are particularly appealing. For example, the common belief in discredited personality types (e.g., the original Myers-Briggs) may arise because their categories provide a welcome causal explanation for behavioral variation, even when they have not been validated.

These errors should not obscure that categorization and attempts to infer causes are essential for cognition and behavior. Indeed, the two key assumptions of essentialism are often functional and appropriate. These heuristics may have developed because they were adaptive for solving practical matters of communication and survival under conditions of uncertainty. Essentialist thinking may provide a cognitively efficient strategy for dealing with complex data, in which thinking in even false categories may be sufficiently fast and functional: Essentialism may be an ecologically rational heuristic (Gigerenzer & Gaissmaier, 2011). Yet this tendency may stymie scientific theorizing, especially in psychology, in which many constructs are indirectly measured and intended to capture complex, multifactorial phenomena.

## Simplicity and Essentialism

Individuals may prefer simpler or abductive explanations. Categories that constitute natural kinds with essences causing their properties and outcomes are the simplest types of categories. Occam’s Razor is widely advocated as good scientific practice and is loosely defined as “the simplest solution is most likely the right one.” Simpler theories reduce the degrees of freedom in an explanatory model, making hypothesis testing more efficient and progress more feasible. Simpler theories may also be preferable because they better describe the structure of nature. Einstein (1934) stated, “The supreme goal of all theory is to make the irreducible basic elements as simple and as few as possible without having to surrender the adequate representation of a single datum of experience.” (p. 165). In sum, individuals may prefer simpler explanations, and scientists may find simpler explanations more useful. However, essentialism is not just a preference for simplicity; it is a set of specific intuitions that can impede progress by facilitating category-based assumptions not supported by the evidence and by generating unfounded causal explanations.

## Pervasive Essentialism in Psychology

Psychological research relies on context-dependent measurements and informal, verbal definitions of phenomena. As a result, psychological research is deeply rooted in how humans think and communicate about categories. Labeling a complex phenomenon can appear as theoretical progress without defining the assumed essence or boundary conditions (Fiske, 2020). These misleading labels can further undermine progress because they obscure the need to better specify mechanisms and cause the neglect of contingent or contextual explanations. Below, we show that diverse research areas across psychology may suffer from searching for essences on the basis of examples from cognitive, clinical, and biological psychology and neuroscience. In each area, we also highlight promising research that is overcoming intuitive roadblocks through the systematic study of variability and contextual influences.

### Clinical psychology

This section explores the use of classification syndromes rather than studying variability in symptoms and the broader medicalization of mental health issues. These criticisms are not novel; presented together, they demonstrate a potential bias toward essentialist explanations.

#### Syndromes versus symptoms

Research in mental health has been shaped for decades by the diagnostic criteria of the *Diagnostic and Statistical Manual of Mental Disorders* (*DSM*). After the latest revision of this manual (*DSM-5*; American Psychological Association, 2013), many researchers and funding bodies began questioning whether its syndromes provided a solid foundation for research. A core issue is that the same diagnostic label (e.g., autism spectrum disorder; ASD) can sometimes arise from very different clusters of symptoms. Thus, a study looking at a group diagnosed with ASD according to the *DSM-5* would mask substantial individual differences. For concepts such as depression, obsessive-compulsive disorder (OCD), and bipolar disorder, the labels, definitions, and diagnostic criteria are constructed, and using such labels may have positive and negative consequences (Borsboom et al., 2011; Boschloo et al., 2015).

The classification into syndromes can be helpful, especially with conditions such as Down syndrome or Williams syndrome, for which there is more validity to the assumption of a coherent set of symptoms with a consistent etiology. Notably, the U.S. National Institutes of Health no longer funds research that relies only on group comparisons based on criteria from the *DSM*. This shift may have taken longer because the syndrome model is intuitively appealing (Hood, 2017). It is important that researchers consider on a case-by-case basis whether a syndrome model is the best way to structure a research program.

We recommend explicitly considering the risk of essentialism when discussing syndrome terms such as ASD or dyslexia or in debating whether autism and Asperger’s are separate conditions. As Duncan (2010) warned, we ought to avoid research questions that are “not about how the world is but about what we call things” (p. 26). In many practical contexts, labels determine access to public services and patient outcomes. Labels serve a critical and practical purpose, but it is easy to forget that a label may not refer to a coherent category with a consistent etiology. However, labels are also useful even when individual outcomes vary. A common etiology might result in different developmental trajectories, particularly for developmental disorders, and thus manifest in different patterns of symptoms (Van de Cruys et al., 2014).

One promising approach to avoiding the intuitions triggered by clinical labels is to apply data-driven methods to symptom clustering. For example, a recent study on executive-function difficulties did not group children on the basis of diagnostic categories but instead identified three clusters of children from their symptoms (Bathelt et al., 2018). These techniques were subsequently used to explore the cognitive (Astle et al., 2019) and neural (Siugzdaite et al., 2020) profiles of these different groups, revealing that the cognitive differences were best predicted by the connectivity of different neural hubs. Although these data-driven approaches are not a panacea for understanding the structure underlying variability in clinical syndromes, they offer substantial promise as tools to falsify intuitive assumptions about the existence of discrete categories (Astle & Fletcher-Watson, 2020). Labels such as depression or OCD are constructed categories whose validity should be tested, and researchers should prioritize the study of variability and context.

#### The essentialist medicalization of mental health

Understanding mental health in terms of syndromes can be seen as part of a wider medicalization of mental health in which clinical conditions are often thought to arise from fundamental biological processes. This view elevates the perceived importance of biomedical treatments such as drugs targeting biological mechanisms. For example, if depression is thought to be caused by a neural imbalance, this directs attention to pharmacological interventions. The extent to which this medicalization is appropriate and helpful is the subject of vigorous debate (Bentall & Beck, 2004; Pilgrim & Bentall, 1999). A recent analysis of approximately 6 million Danish individuals followed over 25 years revealed that comorbidity is the rule rather than the exception. This goes some way toward explaining why attempts to find biological markers for mental illness either through genetics or neuroimaging have largely proved fruitless (Plana-Ripoll et al., 2019). This debate might be biased by intuitive beliefs about essences. For example, there is a massive body of research on the underlying cognitive or neural causes of depression and a particular focus on the serotonin hypothesis, which we return to below. The biomedicalization of mental health may have gained more traction than is justified by the evidence because that interpretation fits essentialist intuitions about categories.

A particular risk with essentialism is the neglect of social and societal context. For example, the extent of mental health issues varies in relation to racial or ethnic segregation (Aneshensel & Sucoff, 1996), social exclusion (C. Morgan et al., 2007), financial hardship (Butterworth et al., 2009), socioeconomic deprivation (Gunnell et al., 1995), socioeconomic status (Lorant et al., 2007), debt (Jenkins et al., 2008), material living standards (Weich & Lewis, 1998a), and subjective financial strain (Weich & Lewis, 1998b). Mental health appears to be worse when inequality is higher, even for high-socioeconomic-status individuals (Weich et al., 2001). We suggest that essentialist intuitions can lead to neglecting contingent or contextual factors in favor of supposedly inherent, underlying cognitive or biological deficits.

**Fig. 1.**
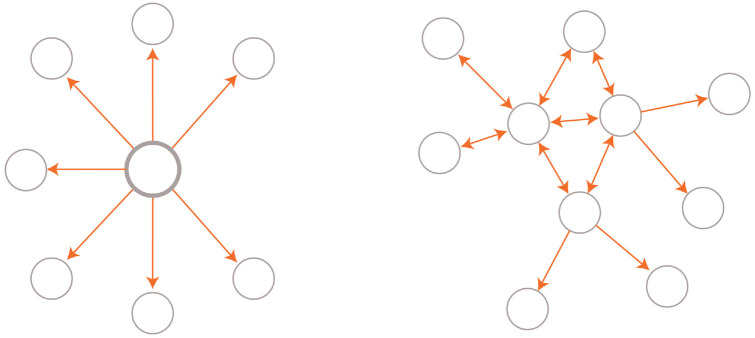
Two competing theories that explain correlations between variables. A unitary force (either real or an illusory essence) causes phenomena (left). A network has variables that cause each other (e.g., symptoms), leading to emergent properties (right).

The neglect of contextual factors in mental health research is likely to impede understanding and therefore treatment and effective public policy. Effective interventions are likely to require treatments through public policy as well as biomedicine. This is especially relevant because mental health issues are increasing in young people (McManus et al., 2016). To understand resilience to mental health challenges, researchers may need to reject the essentialist account of resilience (see, e.g., “We abandon the notion of resilience as an entity here”; Kalisch et al., 2019, p. 1). Instead, resilience can be seen as a dynamic process in a network with multiple factors such as biology, cognitive capacities and skills, and social support. Conceptually, this is like a flock of birds turning without any central control (J. Morgan, 2019). Figure 1 depicts two conceptual models, one with a central essence and causal force and the other without (Fried, 2020).

### Biological psychology and neuroscience

This section explores three examples of how essentialism may influence the interpretation and research focus in biological psychology: the misleading intuitions that (a) single genes and (b) hormones and neurotransmitters are appropriate and useful placeholder essences and (c) the misleading categories that guide neuroimaging research, along with the assumption that neural activity is the cause of those categories.

#### The psychological essence of genes

A huge range of complex psychological traits have heritable variance (Plomin et al., 2016), and genetic contributions to behavioral traits can trigger a range of essentialist intuitions. Essentialism appears to bias scientific reasoning about the contribution of genetics to sexual preference (Ganna et al., 2019). When learning about this relationship, people often assume the existence of a single gene that causes the trait (Dar-Nimrod & Heine, 2011). People make this assumption without a mechanistic understanding of how DNA codes for proteins that drive biological processes. Instead, single-gene explanations may be intuitively appealing because the gene serves as a placeholder essence. In the popular press this tendency is often captured in headlines purporting to have found “the gene for X.”

An education in psychology is not sufficient to overcome the appeal of single genes. It remains profoundly counterintuitive that cognitive and behavioral traits are massively polygenic (depending on hundreds or thousands of genes) and are driven by complex gene-by-environment interactions (Plomin et al., 2016). Genetic contributions to behavior are often socially contingent and more probabilistic and interactive than deterministic (Plomin et al., 2016). Decades of research on single candidate genes were required to disprove the appealing idea that single genes were the correct place to carve nature to understand complex phenotypic and behavioral traits (Border et al., 2019). These findings are slow to be accepted, perhaps in part because they challenge an intuitive explanation based on fixed essences.

#### The psychological essence of hormones and neurotransmitters

All behaviors have a biological basis, and neurotransmitters have distinct functions. However, some hypotheses in cognitive neuroscience link complex psychological categories to single hormones or neurotransmitters. Several of these prominent attempts have resulted in theoretical dead ends, perhaps because of essentialist intuitions that specific hormones or neurotransmitters are the essence of an intuitive category. Despite substantial research expenditure and scientific attention, the latest consensus suggests that there is no direct link between serotonin and depression (Healy, 2015), and oxytocin is not well characterized by compassion or prosocial behavior (Tabak et al., 2019). Sex differences in testosterone are often thought of as innate and natural, and testosterone could be seen as an essentially male hormone, but recent evidence suggests that testosterone levels are also related to specific behaviors such as competition (van Anders et al., 2015) and parenting (Lawson et al., 2017). To the extent that testosterone is seen as an essential and identifying aspect of male biology, this evidence may be overlooked, and the role of testosterone in both genders may be misunderstood.

Linking even well-defined biological processes to poorly defined categories of behavior (e.g., depression, compassion, masculinity) is unlikely to be productive. In contrast, more revealing studies often explore precisely defined variability within a particular task. For example, the ability to make a rapid eye movement to a target on one side of a screen when simultaneously presented with a distracting stimulus on the other side of a screen is associated with concentrations of γ-aminobutyric acid (GABA) in the frontal eye fields (Sumner et al., 2010). Thus, a precisely defined aspect of motor control can be linked to a clear neural correlate. However, it is unlikely that there is a single neural correlate of distractibility because such a term refers to a family of related behaviors for which there is probably not an underlying essence, and GABA is unlikely to be the single cause of variability in these behaviors.

#### Neural activity as an essence in visual neuroscience

In the previous sections we reviewed how genes and neurotransmitters are sometimes assumed to be the causal essences underpinning psychological categories from common language. The pitfalls of essentialism are more apparent in fields such as social psychology, in which it is more evident that constructs such as implicit cognition are elusive and inconsistently defined (Corneille & Hütter, 2020). The risks of essentialist thinking are less obviously a problem in neuroimaging, thus creating a valuable opportunity for evaluating the risks of essentialism. We argue below that neuroimaging theory is being held back by the assumption that a pattern of neural activity is the causal essence of an intuitive category.

Neuroimaging frequently uses constructs such as information, representation, or neural code. These constructs are often used in a manner that obscures rather than clarifies the underlying computational process. Focused on the term “information,” Shannon (1948) made clear that information was not an inherent property of a physical response but rather only a meaningful concept when considered in relation to a specific decoder (receiver). However, interpretations of neuroimaging results rarely make this definition explicit and often implicitly assume that signals decoded by an external observer (fMRI, cell recording, electroencephalogram) are signals that can be decoded by the brain (de-Wit et al., 2016). This assumption underpins much of modern neuroimaging but is rarely explicitly tested, possibly because it taps into an intuition that information is somehow an inherent property of physically decoded signals. It might be obvious that femininity is not inherent to the color pink (Cimpian & Salomon, 2014), but information is also not inherent to the firing of a neuron or a pattern of neural activity. Instead, it is contingent on how that physical difference might be decoded by the rest of the brain. Brette (2019) argued that the representational challenges of sensory processing and the complexity of the dynamic brain mean that the coding metaphor is actively misleading for thinking about how the brain works. Recognizing these challenges can conflict with essentialist intuitions. The notion that some aspect of neural activity might represent categories such as love (Bartels & Zeki, 2000) or a Christmas-spirit network in the brain (Hougaard et al., 2015) are transparently problematic. However, the intuition that activity in the brain somehow acts as a placeholder essence for categories from common language is also evident in the attempts to interpret canonical findings in visual neuroscience such as the single-cell response to edges and the fMRI response to faces.

Edges are a particularly interesting example because unlike some concepts in psychology, physical discontinuities in luminance (edges) are a discoverable feature of the external world, and explicit computations can show that luminance transitions are informative units of representation in models that attempt to decode (recognize) different objects. The observation by Hubel and Wiesel (1959) that cells in V1 fire when an edge is presented to their receptive field is foundational to the understanding of how the visual system works. However, although luminance discontinuities exist outside the brain, the exact boundary conditions that constitute an edge are not specified. As with the Ship of Theseus, if one takes a given luminance threshold and increases or decreases it, there is no objectively defined moment at which that luminance boundary becomes an edge. Thus, the idea that V1 cells are edge detectors is problematic because nothing in the physics can reveal what is or is not an edge, leading Koenderink (2012) to observe that “it remains unclear what precisely is meant by an ‘edge’—apart from being what edge detectors detect” (p. 35). This problematic circularity reflects a wider problem in thinking about the inherent nature of concepts that is evident across psychology, perhaps most aptly captured in this well-known operational definition from Boring (1923): “Intelligence is what the tests test” (p. 35).

However, visual neuroscientists are now well aware that the category of an edge might not provide a solid foundation for research. Indeed, it is now clear that the firing rate of V1 cells is tuned not to simple edges but to certain patterns of spatial-frequency distributions. This is the point at which the more pernicious intuitions kick in, namely that the firing of V1 neurons in response to this stimulus feature has the inherent property of representing that stimulus, even if the stimulus is now more clearly specified. Despite the half-century since Hubel and Wiesel (1959), this coding assumption has never been directly tested. More specifically, it is not definitively known whether the firing rate of neurons in V1 communicates information to other areas of the brain about the existence of luminance boundaries. For example, it could be the timing at which those neurons are firing that actually communicates information (Schyns et al., 2011). Some aspects of visual recognition happen so fast that perhaps the timing of the first spike in response to a stimulus onset communicates information to other areas of the brain (Thorpe et al., 2001). This has important implications, because if information is conveyed via precise timing rather than the firing rate, then techniques such as fMRI that have poor temporal resolution would be unable to detect information processing in the brain (de-Wit et al., 2016). As in other areas of psychology, one reason these assumptions have been overlooked may be because the idea that information is an inherent property of a physical stimulus is congruent with essentialist intuitions.

Even if the firing rate of a neuron does prove to be the channel through which information is conveyed from V1 to other areas of the brain, there are problems with the idea that activity in V1 represents the essence of this stimulus category. Studies have highlighted that V1 responses are not easily predicted solely on the basis of input stimuli (Murray et al., 2006). In fact, when presented with more complex images, V1 cell responses do not seem to reflect the presence of edges in a straightforward way (Olshausen & Field, 2005). This finding has led some to argue that the response to isolated edges (as identified by Hubel and Wiesel) might be a quirk and that the response of V1 cells can be understood only when presented with more complex stimulus patterns (Alexander & Van Leeuwen, 2010), which highlights the idea that the study of how neurons behave in different contexts can help to falsify essentialist intuitions. One of the most surprising of these contextual responses is observed in mice, in which the firing rate of nearly half of the cells in V1 is influenced not only by the presence of visual stimulation but also by whether or not the animal is running (Saleem et al., 2013).

In sum, the concept of edges is intuitive but potentially misleading and is just one example of the search for the neural essence of categories from common language. The human ventral visual system has been mapped out in terms of the following intuitive categories: the fusiform face area (Kanwisher et al., 1997), extrastriate body area (Downing et al., 2001), visual word form area (McCandliss et al., 2003), object area (Malach et al., 1995), motion area, color area (Zeki et al., 1991), and perhaps even tool (Chao et al., 1999) and hand (Bracci et al., 2012) areas. There are reasonable empirical reasons for conceptualizing the organization of the human visual system in this way, such as the observation that some neuropsychology patients selectively cannot recognize faces (Farah, 1990) or the results from imaging studies highlighting the differential activity of some areas in processing some types of stimuli (Zeki et al., 1991). Labeling the human visual system with intuitive concepts is an understandable approximation. However, reflecting back on Gelman’s two-part definition of essentialism, these approximations also trigger the intuitions that these categories are real and constitute a sound basis for carving nature at its joints and that that neural activity in these areas is a primary cause.

Ill-defined categories generate recurring theoretical problems. For example, debates about whether a particular area is specialized for faces will be hard to resolve given that the familiar label does not specify what is or is not a face or whether activity in response to configurations of stimuli similar to faces counts as evidence for or against a specialization for faces (Brants et al., 2011). Likewise, in identifying the function of an area labeled as processing objects, should this area respond only to patterns of visual input that correspond to a lexical entry (an object that can be named) or to any coherent shape such as a blob with no semantic or lexical meaning (Malach et al., 1995)? Labels such as objects, faces, tools, or hands ultimately offer a first-pass approximation of the distribution of functions across the visual system, but researchers are unlikely to ever resolve debates about whether areas of the brain are specialized for categories that represent a word from everyday language because it may have no unitary essence (Duncan, 2010). Visual neuroscience is now progressing from the phase of labeling brain areas to developing explicit computational models of the processing steps involved in visual perception. Rather than trying to map intuitive, undefined terms from common language onto areas of the visual cortex, researchers are quantitatively comparing the responses at different stages of the human visual system with the responses at different stages within computational models (Kriegeskorte, 2015). As this research agenda progresses, the intuitive labels previously used for different areas in the visual system may be replaced with explicit computational processes.

### Cognitive science

In this section, we suggest that the deceptive appeal of essences is also widespread in cognitive science. The shift from behaviorism to cognitive science depended on the ability to assume the existence of processes or mechanisms underlying observable behavior. This revolutionary step also created space for positing vague cognitive essences that sometimes hamper scientific progress. When new terms are introduced but not precisely defined, theoretical progress may be slowed. We use “attention” as an example of a label that can easily be misunderstood.

#### Assuming constructs are unitary

Attention is one of the most widely used constructs in cognitive science. As noted by Newell (1973) in his landmark article, much research in cognitive science tries to formalize questions in terms of whether a certain cognitive process requires attention or whether it is attentive or preattentive. Despite William James’s formulation that “everybody knows what attention is,” a lack of progress indicates that perhaps no one knows what attention is (Hommel et al., 2019). Attention is currently used to refer to qualitatively different selection processes, and many attention researchers in neuroscience acknowledge this breadth (Desimone & Duncan, 1995). Attention may be an emergent property of the way in which neural representations compete for cortical processing and how that processing is biased (Duncan, 2006). However, Hommel et al. (2019) argued that more progress would be possible if the term attention were dropped in favor of models that account for specific, behaviorally relevant selection processes and the many systems that implement them. However, abandoning the term attention risks obscuring that different selection mechanisms are part of the same underlying goal to process information efficiently and usefully across different sensory modalities. Without an overarching conception of this goal, one might also miss commonalities in the selection mechanisms used across different systems (Duncan, 2006). We suggest that psychologists should be aware of the biases their cognitive processing brings to labels such as attention and explicitly state and evaluate essentialist assumptions. Although some readers might understand that attention refers to an emergent property, even expert readers may assume that attention refers to a definable unitary essence.

#### Assuming common variance between tasks

Research on attention is just one example of the general challenge of trying to develop labels that might help clarify the commonalities between related processes while not implying underlying inherent essences. This challenge is unlikely to be entirely solved by using or avoiding certain terms. Cognitive science has recently increased its focus on individual-differences research that directly tests whether different constructs measure the same underlying mechanism. For example, different theory-of-mind tasks turn out to be minimally associated (Warnell & Redcay, 2019). Likewise, in perceptual cognition research the tasks that measure global versus local bias unexpectedly show minimal associations (Chamberlain et al., 2017). In visual neuroscience research on dyslexia, a range of tasks has been used to assess magnocellular function, but these tasks also have minimal associations (Goodbourn et al., 2012). In each of these instances, this research indicates a healthy debate about the construct validity of different tasks. Here, the intuitive appeal of essences may decrease the perceived necessity of empirically evaluating whether variability across different tasks is driven by a common mechanism.

The tools for this form of empirical validation were among the first methodological innovations in psychology. Spearman helped to develop factor analysis to test for the existence of a general problem-solving ability (*g*). Perhaps greater progress could be made in psychology by following Spearman’s model to first establish that an underlying construct is represented by a single factor measured across different tasks before testing for associations with other constructs or outcomes. Even when there is evidence for a positive correlation (well replicated in the case of *g*), this may not reflect a single underlying latent process. Factor analysis can help to falsify the assumption of a common cause, but positive correlations do not prove such a common cause exists (van der Maas et al., 2006).

#### Assuming universality

The pattern of positive correlations evident in Spearman’s *g* was recently replicated across 31 non-Western nations (Warne & Burningham, 2019). Diverse populations and cultural contexts are also necessary for establishing the validity of factors, but psychologists often rely on narrow, unrepresentative samples. Recall the argument from above that essentialist intuitions may detract from contingent or contextual causes. Cognitive science is also vulnerable to the assumption that observations in one setting reflect inherent and stable human characteristics. Most behavioral science is conducted with WEIRD (Western, educated, industrialized, rich, and democratic) participants (Henrich et al., 2010; Rad et al., 2018). A classic research question in cognitive science is whether spatial-cognition abilities might be different in males and females. This difference was replicated in numerous samples in the United States and United Kingdom and could easily be assumed to be a stable and universal feature of human sex differences. However, in a recent study that analyzed data from 2.5 million people, the size of the sex differences varied substantially across countries (Coutrot et al., 2018). The differences between sexes were correlated with gender inequality and may be explained by different opportunities to gain experience in spatial navigation (e.g., driving a car).

Using mostly WEIRD samples can lead to overgeneralization. The practical difficulties of recruitment are a major cause of narrow sampling, but non-WEIRD samples may be further neglected because of intuitive essences. Most studies use the data from limited samples “in an unreflective way to make inferences about humans in general” (Rad et al., 2018, p. 11401). If one assumes a Platonic notion of a fundamental essence of humanity that is nearly universal across cultures and contexts, there is no need to consider limitations of generality. Some features and processes are universal in humans, but there is less mechanistic evidence for the concepts and processes in psychology than those in biology such as aging. We agree with DeJesus et al. (2019) that “when participants from non-WEIRD samples perform differently, this is often described as abnormal or problematic” (p. 18371). Ascribing universality should be a topic of careful reflection and debate, toward which we should be investing heavily in the empirical study of cross-cultural comparisons and declaring sensible and explicit targets for generalization (Simons et al., 2017). Fortunately, new large-scale research collaborations such as the Psychological Science Accelerator are collecting more cross-cultural data (Moshontz et al., 2018), and platforms such as Prolific are making it easier to collect samples in different countries.

## Strategies for Reducing Essentialism

In this section, we offer four strategies for revealing and managing essentialist intuitions. We developed these recommendations on the basis of the function and consequences of essentializing and by drawing on positive examples of research that help to mitigate essentialist intuitions.

Early scholars also wrote about reducing essentialist tendencies in psychology. B. F. Skinner quoted Newton as saying: “To tell us that every species of thing is endowed with an occult specific quality by which it acts and produces manifest effects is to tell us nothing” (1971/2002, p. 9). Skinner continued: “Behavior, however, is still attributed to human nature” (1971/2002, p. 13). In short, the extreme behaviorist solution is to measure only behaviors, contexts, and outcomes. We think there is value in attempting to make inferences about internal mental processes, but with the huge increase in cognitive labels between 1940 and 2010 (Whissell et al., 2013), psychology may need to develop more nuanced strategies to mitigate essentialist intuitions. The recommendations we offer below (see also Table 1) are not yet empirically validated and offer a starting point that will require testing and refinement.

**Table 1.** Strategies for Reducing Essentialism

1. Transparently discuss what is known about mechanisms
2. Evaluate contextual and contingent explanations
3. Explicitly test phenomena for a common underlying cause
4. Consider using unfamiliar construct labels

### Strategy 1: transparently discuss what is known about mechanisms

The construction of taxonomies by which phenomena are categorized is an important part of the scientific method, particularly in the biological sciences. The past 50 years of psychology show that researchers labeling constructs could be more explicit about the lack of a causal explanation underpinning that category. A prominent example is the field of “cognitive biases,” in which human behavior is catalogued as a seemingly never-ending list of deviations from a context-free rationality. That said, the attempt to label different phenomena as manifestations of the same effect (e.g., confirmation bias) could help to develop hypotheses about the mechanisms that underlie these behaviors and ultimately inform computational models. For example, Rollwage et al. (2018) looked at the seemingly vague concept of dogmatic intolerance and found that this mode of thinking can be modeled in terms of a specific deficit in metacognition. We have also argued that it is useful to examine psychological theories for essentialist thinking, but the exact mechanisms of essentialism are unknown. There may be no core essence to essentialist thinking; it may arise as an emergent property of various aspects of information processing. It also remains unclear whether it is most useful to characterize essentialism as a unitary process or the sum of related components (Gelman et al., 2004). Regardless, essentialism is a useful label that helps reveal a family of related challenges in psychological theorizing (Gelman, 2005a, 2013; Medin & Ortony, 1989). In sum, explicitly describing what is known about mechanisms may reveal essentialist assumptions and ultimately improve falsifiability.

### Strategy 2: evaluate contextual and contingent explanations

When an empirically quantifiable phenomenon is identified in human behavior, one could look for potential explanations in terms of stable or inherent properties or for contingent and contextual circumstances. The underlying causal mechanisms could lie on either side, but essentialist intuitions could make inherent mechanisms more appealing and lead to a neglect of contingent or contextual effects. The goal of this strategy is to prioritize descriptive variability across contexts. For example, research has suggested that self-control could be measured with a simple marshmallow task that assesses whether a child will wait to eat one treat to receive a second (Mischel, 1974).

This test is useful in offering a clearly quantifiable phenomenon. Furthermore, the task seems important because individual differences predict a variety of important outcomes later in life (Mischel et al., 1988). It becomes tempting to assume that this task measures an inherent or stable cognitive mechanism, for example, cognitive control, that explains performance on this task and outcomes later in life and to use this explanation in guiding education or public policy. However, the essentialist temptation overlooks other explanations. What differs between children may not be a cognitive ability to regulate their behavior but rather a belief about the reliability and predictability of the world (Kidd et al., 2013). A child could have excellent self-control, but if previous experiences lead the child to be unsure that the second (promised) marshmallow will arrive, then it is rational to eat the first. Subsequent research highlighted that this belief, and the contexts that cause variability in this belief, may be the key to why this task predicts later outcomes. The original explanation in terms of cognitive mechanisms may feel intuitive. However, this intuition risks narrowing the search for alternative explanations (Doebel, 2020).

The key goal is to measure variability across contexts to attempt to falsify a unitary explanation and to determine constraints of generality. As discussed above, apparent differences in spatial cognition across genders have been shown to result from contextual variability. The key advance in resolving these apparent differences was systematically exploring variability in the phenomenon and boundary conditions (see also Scheel et al., 2021). Rather than assuming an inherent essence, Darwin systematically measured both variability across contexts and the factors that might cause that variability. This might seem like an obvious lesson. However, the enduring reliance on WEIRD samples and universal explanations suggest that there remains a need in psychology to systematically measure variability across cultures and contexts. Scientific societies, journals, and funding bodies are starting to prioritize cross-cultural comparisons, and these efforts may help to reveal essentialist intuitions.

### Strategy 3: explicitly test phenomena for a common underlying cause

We highlighted examples above in which empirical tests for putative constructs discovered that different tasks turned out not to measure the same underlying mechanism (theory of mind, local/global, magnocellular). These tests are foundational in theory building, but in these cases the disconfirmations came only after extensive research using these tasks and assuming a common underlying mechanism. Likewise, despite decades of research using the categories from the *DSM*, progress has been slow on data-driven approaches to how people and their characteristics are clustered. We recommend that psychologists more explicitly test (a) the assumption that different tasks depend on a common factor, (b) how participants cluster into distinct groups (Astle et al., 2019), and (c) how the groups overlap (Hanel et al., 2019).

### Strategy 4: consider using unfamiliar construct labels

It is seductively easy to use familiar words that lack a precisely defined meaning (Rozenblit & Keil, 2002; Wittgenstein, 1953). Labels such as “intelligence” or “attention” might seem like well-specified theoretical constructs not because of an agreed-on scientific definition but because they resonate with intuitive essences. One effective strategy may be building explicit computational models (Oberauer & Lewandowsky, 2019). However, many areas of psychology lack formal theories. In these areas, verbal explanations are particularly susceptible, and further strategies would be helpful.

Emotion research is one of the earliest areas to explicitly discuss the issues of common language labels and essentialist thinking (Barrett, 2006). One promising example of avoiding the fuzzy familiarity of common words is labeling an emotion with an unknown term, such as *kama muta* instead of “being moved” (Fiske, 2020). This choice avoids the effortless and consequential associations English speakers have with being moved and instead allows the researchers to document variability across individuals and contexts and more explicitly test whether the construct is deep and invariant. An unfamiliar label may avoid biasing the search space to familiar triggers or reactions and reduce the risk of assuming categories are natural kinds.

In practice, researchers can reflect on how much a construct relies on a term taken from common language. The more common and intuitive the word, the greater the danger of operating without a formal definition. For example, in research on face perception, when developing a theory that a part of the brain is specialized for faces one could specify in advance what counts as a stimulus that should be processed in such an area (e.g., cartoon faces, animal faces, upside-down faces). Without such specification, essentialist intuitions may bias the interpretation of results (e.g., correlations with regional brain activity).

Spearman adopted the unfamiliar label strategy when naming the common variance in performance between different tasks. Rather than using a word from common language such as intelligence, Spearman referred to the common variance as *g*. Any term can lead to essentialist misunderstanding, but avoiding terms for common usage may help, as with the deliberately unfamiliar emotion label *kama muta* (Fiske, 2020) or System 1 and System 2 thinking (Kahneman, 2011). When Kahneman popularized this dual-process theory, he stated that the distinction is a useful fiction that provides ways of thinking about cognition and that the terms were not to be taken seriously as distinct cognitive or neural systems. Labeling these terms System 1 and System 2 may help in communicating efficiently, and strategies for reducing essentialist inferences may reduce the likelihood that researchers would invest resources looking for System 1 and System 2 in the brain. Future researchers could investigate whether the neutral labeling strategy reduces essentialist intuitions (and which ones).

## Conclusion

Assuming unitary, causal essences appears pervasive and problematic in psychological theory at least across cognitive, clinical, and biological psychology and neuroscience. Behaviorism recognized the pitfalls of making inferences about internal psychological processes. However, rather than returning to behaviorism we advocate a psychological science that pays more attention to how human cognition shapes categorization and theorizing. “Everything is vague to a degree you do not realize till you have tried to make it precise, and everything precise is so remote from everything that we normally think” (Russell, 1919, pp. 161–162).

## Transparency

*Action Editor:* Laura A. King

*Editor:* Laura A. King

*Author Contributions*

L. de-Wit developed the idea, C. Brick and L. de-Wit rebuilt the argument, and C. Brick rewrote the manuscript. All of the authors made substantive contributions to the argument, narrative, and examples and approved the final manuscript for submission.
